# Repeated and On-Demand Intracellular Recordings of Cardiomyocytes Derived from Human-Induced Pluripotent Stem Cells

**DOI:** 10.1021/acssensors.2c01678

**Published:** 2022-09-27

**Authors:** Jihyun Lee, Tobias Gänswein, Hasan Ulusan, Vishalini Emmenegger, Ardan M. Saguner, Firat Duru, Andreas Hierlemann

**Affiliations:** Tobias Gänswein — *Bio Engineering Laboratory, ETH Zurich, 4058 Basel, Switzerland*;; Hasan Ulusan — *Bio Engineering Laboratory, ETH Zurich, 4058 Basel, Switzerland*; Vishalini Emmenegger — *Bio Engineering Laboratory, ETH Zurich, 4058 Basel, Switzerland*; Ardan M. Saguner — *Cardiac Electrophysiology Division, University Heart Center Zurich, University Hospital Zurich, 8091 Zurich, Switzerland*; Firat Duru — *Cardiac Electrophysiology Division, University Heart Center Zurich, University Hospital Zurich, 8091 Zurich, Switzerland; Center for Integrative Human Physiology, University of Zurich, 8057 Zurich, Switzerland*

**Keywords:** high-density microelectrode arrays, intracellular recordings, induced pluripotent stem cells, cardiomyocytes, electroporation

## Abstract

Pharmaceutical compounds may have cardiotoxic properties, triggering potentially life-threatening arrhythmias. To investigate proarrhythmic effects of drugs, the patch clamp technique has been used as the gold standard for characterizing the electrophysiology of cardiomyocytes *in vitro*. However, the applicability of this technology for drug screening is limited, as it is complex to use and features low throughput. Recent studies have demonstrated that 3D-nanostructured electrodes enable to obtain intracellular signals from many cardiomyocytes in parallel; however, the tedious electrode fabrication and limited measurement duration still remain major issues for cardiotoxicity testing. Here, we demonstrate how porous Pt-black electrodes, arranged in high-density micro-electrode arrays, can be used to record intracellular-like signals of cardiomyocytes at large scale repeatedly over an extended period of time. The developed technique, which yields highly parallelized electroporations using stimulation voltages around 1 V peak-to-peak amplitude, enabled intracellular-like recordings at high success rates without causing significant alteration in key electrophysiological features. In a proof-of-concept study, we investigated electrophysiological modulations induced by two clinically applied drugs, nifedipine and quinidine. As the obtained results were in good agreement with previously published data, we are confident that the developed technique has the potential to be routinely used in *in vitro* platforms for cardiotoxicity screening.

**Figure:**
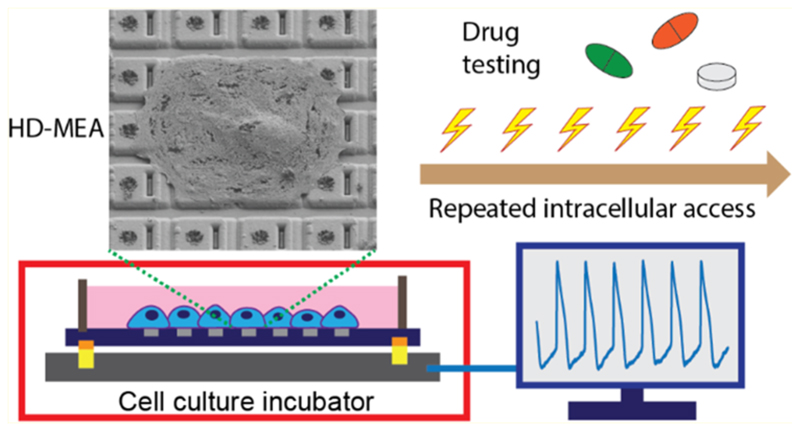

Cardiovascular diseases remain the leading cause of death in Western countries^1^ so that advanced disease models and testing platforms are needed to find and develop optimal prevention and treatment strategies. In addition, drugs that are not targeted at the cardiovascular system need to go through cardiac risk assessment, as they may have cardiotoxic or proarrhythmic features.^2^ Numerous drugs have been with-drawn from the market due to reports of cardiotoxicity or potentially life-threatening off-target effects,^3^ such as QT-interval prolongation, myocardial infarction, and torsades de pointes (TdP) tachycardia. For instance, doxorubicin, a topoisomerase II inhibitor, used for chemotherapy of cancers, has well-known side effects on the heart, such as arrhythmia and cardiomyopathy.^4^ These side effects may endanger the life of the patient and preclude a successful completion of the treatment. Unfortunately, drug-induced cardiotoxicity is often only detected after 5–10 years of drug development in clinical trials, or even after approval and use with larger patient groups. In addition to life-threatening risks for patients, there are substantial financial losses for pharmaceutical companies upon withdrawal of medically efficient, yet cardiotoxic drugs.

In the past, drug responses and cardiotoxic effects were evaluated in animal or *in vitro* models, such as Chinese hamster ovary (CHO) cells and human embryonic kidney 293 (HEK293) cells that express specific ion channels. Although animal models provide insights into multiorgan interactions, they have several limitations, including interspecies differences, low throughput, high costs, and ethical concerns.^5–7^ CHO and HEK293 cells expressing a single-ion-channel gene are limited in recapitulating the properties of human cardiomyocytes (CMs).^8^ Importantly, genetic and physiological discrepancies between such models and human heart cells challenge the translation of research outcomes to the clinical setting. The advent of human-induced pluripotent stem cell (iPSC) technology and protocols to differentiate them into CMs enables to perform *in vitro* drug screening and risk assessment on human cells under physiological conditions that resemble those of the human heart.^9^

The patch clamp technique has been the gold standard for measuring action potentials (APs) and ionic currents across the cell membrane *in vitro*^10^ and to investigate proarrhythmic effects of drugs. Although the originally manual and individual patching of cells provides precise measurement of voltage and current dynamics, the intense training that is needed for applying the patch clamp techniques and the low-throughput limit its application for testing on a large scale. Another drawback of the technique is that it is highly invasive. The patch pipette ruptures the cell membrane over an area of approximately 1–2 *μ*m in diameter upon intracellular access. The membrane remains open for 30–60 min, while the pipette solution mixes with the cell cytosol^11^ causing gradual cell death.^12^ The use of automated patch clamp (APC) techniques allows for intracellular recordings of a few hundred suspended cells in parallel by aspirating the cells to orifices and subsequent cell opening,^2,13^ which makes APC suitable for high-throughput screening. However, for APC, CMs need to be provided in single-cell suspension, which is different from physiological conditions in the human heart, where CMs form a network and beat synchronously. Fluorescence-based imaging techniques, such as voltage-sensitive dyes or voltage-sensitive fluorescent proteins, are comparably noninvasive and can be used to perform recordings of many cells at a time. Nevertheless, there are limitations concerning cytotoxicity, phototoxicity, photobleaching, or limited efficiency of protein expression.^12,14,15^

The main advantage of applying microelectrode arrays (MEAs) or, specifically, high-density microelectrode arrays (HD-MEAs) is that it enables long-term and noninvasive extracellular recordings of hundreds to thousands of cells^16–19^ at high spatiotemporal or even subcellular resolution.^20,21^ Following a relatively simple procedure, several studies reported on utilizing MEAs to assess the proarrhythmic effects of drugs on human iPSC-derived CMs.^22–24^ Although extracellular recordings with MEAs have shown a great potential for large-scale cardiotoxicity assessment, extracellular measurement alone is limited in capturing the full dynamics of membrane processes and associated electrical potential alterations in comparison to intracellular AP recordings.^25^

Over the past years, numerous reports^26–32^ have shown that MEAs can be used to measure not only extracellular signals but also intracellular-like signals, by transiently opening nanopores in the cell membrane. Advances in nanotechnology improved the coupling of plasma membrane and electrodes by integrating 3D nanostructures, such as nanopillars,^14,33–35^ nanowires^36–39^ nanocrowns,^40^ nanobranches^41^ gold-mush-rooms,^42^ and nanovolcanos,^32,43^ all of which enabled spontaneous intracellular access or access via electroporation. Although nanostructured electrodes promote membrane penetration or are advantageous for being engulfed by the cell membrane, the fabrication of such nanostructures requires intensive and expensive cleanroom processing, which, to date, impeded application in large-scale drug screening and cardiotoxicity assays in the pharmaceutical industry. In addition, nanopillar or nanowire electrodes significantly attenuate voltage signals due to the high electrode impedance in comparison to planar electrodes.^25^ MEAs featuring planar electrodes have been demonstrated to record intracellular-like signals after optoacoustic poration^31^ or electroporation.^26–28^

Optoacoustic poration enabled to record intracellular signals of a few thousand CMs, but this method requires an integration of the MEA with a laser and microscopy system, which renders the setup bulky and complex. While electroporation using commercially available MEAs yielded intracellular-like recordings, the number of CMs that could be measured at a time was limited to a few tens. Moreover, substantial cell-to-cell variations of the electrophysiological waveform characteristics of iPSC-derived CMs^44^ require that electrical signals—after drug exposure—are compared to baseline signals of the same CMs before drug exposure and that data from large numbers of cells are collected and analyzed to exclude spurious effects and avoid bias. In addition, the evaluation of CM network and signal propagation parameters greatly benefits from having a large sensor density and many data points. Finally, the ability to collect large numbers of data points at once—without having to reproduce the assays many times to achieve the desired statistical power—constitutes a big advantage for drug testing. Repeated intracellular access at a large scale is desirable for reliable cardiotoxicity testing with iPSC-derived CMs, and the effects of (repeated) electroporations on the electro-physiology of the CMs need to be investigated.

Therefore, we developed an electroporation-based method for an existing HD-MEA^16^ that enabled us to repeatedly record intracellular-like signals of hundreds of iPSC-derived CMs. The method does not require the fabrication of nanostructures or the use of complex setups to achieve intracellular-like recordings. It relies on the use of electrical stimuli that porate the cell membrane only briefly and can be applied multiple times over an extended period of several hours. The CMs were observed to recover quickly and could be repeatedly porated for a series of intracellular-like measurements. The compactness and simplicity of the setup enabled us to perform experiments in a standard, humidified CO_2_ incubator, which is a great advantage for long-term cellular assays. We repeatedly electroporated hundreds of iPSC-derived CMs at the same electrode locations within defined time intervals and investigated the corresponding effects on extracellular and intracellular-like signals. We demonstrated that the proposed technology may help to set up a cardiotoxicity assay platform to assess short- and long-term proarrhythmic effects of preclinical drugs. The potential to detect cardiotoxicity at an early stage of drug development will promote patient safety and will be beneficial for cardiac testing in the pharmaceutical industry.

## Experimental Section

### Reagents and Materials

Quinidine (Cat. 22600)’ nifedipine (Cat. N7634), dimethyl sulfoxide (DMSO, Cat. D4540), and human fibronectin (Cat. FC010) were purchased from Merck KGaA (Darmstadt, Germany). Fetal bovine serum (FBS, Cat. 10270106) and Intracellular Fixation Buffer (Cat. FB001) were purchased from Thermo Fisher Scientific (Massachusetts). Y-27632 (Cat. 72302) was purchased from STEMCELL Technologies (Cologne, Germany) and dissolved in sterile deionized water to prepare a 10 mM stock solution. During drug experiments, the concentration of DMSO in the culture medium did not exceed 0.1%. Serum-free CM culture medium used in this study was RPMI with GlutaMAX and HEPES (Cat. 72400021), supplemented with B27 (Cat. 17504044) and PenStrep (Cat. 15070063), all purchased from Thermo Fisher Scientific (Massachusetts).

Frozen vials of human iPSC-derived CMs were purchased together with iCell Plating Medium and iCell Maintenance Medium from Fujifilm Cellular Dynamics International (Wisconsin). The cells were thawed and cultured following the manufacturer’s guidelines. The healthy-donor-derived iPSC line, CW30318CC1, was obtained from the CIRM hPSC Repository funded by the California Institute of Regenerative Medicine (California). This cell line was differentiated into CMs following a previously published protocol.^45^

### Plating of iPSC-Derived Cardiomyocytes on High-Density Microelectrode Arrays

Complementary metal-oxide semiconductor (CMOS)-based HD-MEAs^16^ were used to record extracellular and intracellular signals of CMs. The HD-MEA accommodates 26,400 electrodes in an area of ∼4 × 2 mm^2^. The data were sampled at 20 kHz. The switch matrix scheme within the HD-MEA was used to route electrodes to 1024 readout channels for simultaneous readout and to 32 stimulation/poration units. We used and compared two types of electrodes: standard 5.5 × 9.3 *μ*m^2^ rectangular electrodes and smaller circular electrodes with a diameter of 4 *μ*m, all of which were spaced at 17.5 *μ*m center-to-center pitch.

**Figure 1:**
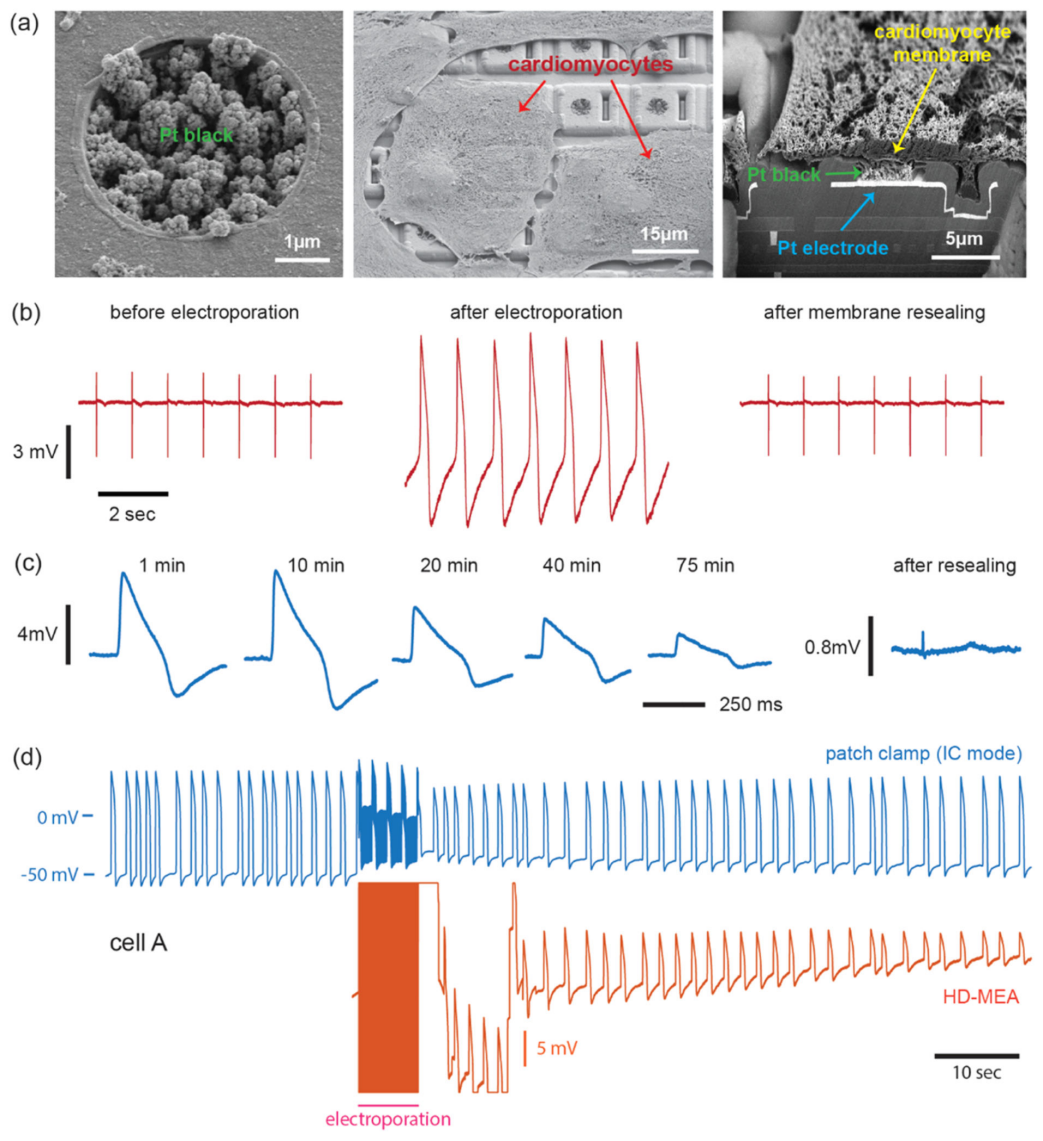
(a) SEM images of Pt-black electrodes and iPSC-derived CMs on the HD-MEA show that the cell membrane conforms well to the porous electrode surface. (b) Extracellular signal recorded from an electrode before electroporation. Immediately after electroporation, an intracellular-like signal with an amplitude of approx. 10 mV was recorded. After membrane resealing, extracellular signal appeared again (from left to right). (c) Long intracellular-like recording from a CM, showing AP waveforms and their changes over time. The signal transited back to extracellular signals after membrane resealing (recovery). (d) Simultaneous recording obtained from the same cell: intracellular recordings by whole-cell patch clamp in the current clamp (IC) mode and intracellular-like recordings obtained by the HD-MEA upon electroporation. The CM continued beating during and after the electroporation as can be seen in the patch recording.

Pt-black was electrodeposited on the bright Pt electrode surface following a previously published protocol^46^ (Figure 1a). All electrodes were connected and a static current of 530 *μ*A was applied for 40 s for the 5.5 × 9.3 *μ*m^2^ electrodes and for 12 s for the 4 *μ*m diameter electrodes in hexachloroplatinate solution.

Before plating iPSC-derived CMs, HD-MEAs were sterilized in 70% ethanol for 10 min and rinsed three times with sterile deionized water. The electrode array was coated with human fibronectin at a concentration of 50 *μ*g/mL and incubated at 37 °C for 1 h. CMs were thawed quickly in a 37 °C water bath, and 30,000 cells were plated on each of the fibronectin-coated HD-MEA electrode areas. Plated cells were allowed to settle in a humidified, 5% CO_2_ incubator at 37 °C. To optimize the electroporation parameters and protocols we used CMs differentiated from human iPSCs in our lab. In this study, a total of 23 cultures from five plating preparations were used. Serum-free CM culture medium, supplemented with 20% FBS and 10 *μ*M Y-27632, was used to plate these CMs. For drug experiments, we used human iPSC-derived CMs (iCell Cardiomyocytes, Cat. R1057), purchased from Fujifilm Cellular Dynamics International (Wisconsin), which are commercially available and have been widely used for drug testing in the literature.^9,22,27^

### Electroporation and Measurements

All extracellular and intracellular measurements were performed in a humidified, 5% CO_2_ incubator at 37 °C. HD-MEA chips were mounted on MaxOne Recording Units (MaxWell Biosystems AG, Zurich, Switzerland) for data acquisition and control of electroporation.

Data acquisition and electroporations were performed using the MaxLive Software (version 19.2.27, MaxWell Biosystems AG, Zurich, Switzerland) in conjunction with a custom-written Python script for API controls. For electroporation, the selected electrodes were connected to on-chip stimulation/poration units, The core of each unit was a class-AB opamp, capable of driving loads as large as 10 nF, while maintaining a low static power consumption (for details, see ref 16). For voltage stimulation, the circuit was configured as an inverting amplifier with low output impedance.^16^ We applied rectangular biphasic voltage pulses of 1-2 V peak-to-peak amplitude (Vpp) and 50 *μ*s to 1 ms phase duration at 1 ms interpulse intervals (IPIs). The poration duration was maximally 35 s. Although the applied voltage amplitude exceeded that of the water window in some instances, the frequency of the AC signal was large enough (333 Hz to 10 kHz range) to not produce electrolysis, and we did not observe any signs of gas evolution. After delivery of the respective voltage pulses, electrodes were disconnected from the stimulation units to perform the voltage recordings. Up to 990 electrodes could be simultaneously connected to stimulation units to perform voltage stimulation.

### Simultaneous Electroporation and Whole-Cell Current Clamp Patch Measurements

To record intracellular signals of CMs with a patch pipette simultaneously with intracellular-like signals through the HD-MEA, we combined the HD-MEA setup with a patch clamp setup and performed recordings outside the incubator. Two data streams from the simultaneous recordings were synchronized by aligning periodic pulses which were sent to both setups. All measurements reported here were obtained from interconnected and spontaneously beating cardiomyocytes. The culture on the HD-MEA was perfused at 37 °C throughout the experiment with RPMI 1640 with GlutaMAX with HEPES. The resistance of the patch pipettes was 5–7 MΩ. The patch pipette solution contained (in mM): KCI (140), MgCl_2_ (1), EGTA (5), HEPES (10), Mg-ATP (5), pH adjusted to 7.2 with KOH.^47^ We recorded APs of the CMs in the whole-cell current clamp mode (Multiclamp 700B, Molecular Devices, California). The data were low-pass filtered at 10 kHz and sampled at a rate of 20 kHz (Digidata 1440A, Molecular Devices, California). The pClamp suite was used as the recording software (version 10.7.0.3, Molecular Devices, California). The data were not corrected for the liquid junction potential, which was approximately 3.1 mV. During whole-cell patch recording, the same cell was electroporated through the HD-MEA, and intracellular-like signals were recorded simultaneously by the patch pipette and the HD-MEA electrode.

### Measurements of Drug Responses

Following the manufacturer’s protocol, iCell Cardiomyocytes (Fujifilm Cellular Dynamics International, Wisconsin) were plated on HD-MEAs and kept in a humidified incubator for at least 10 days. After connecting the HD-MEA chips to the recording units in the humidified incubator, CMs were allowed to equilibrate for at least 30 min. Before drug administration, baseline measurements were performed three times over a time span of 2 h. The reason for this procedure was to rule out the possibility that the electrophysiological perturbation observed after drug administration would arise from CMs themselves and not from the drugs. Stock solutions of quinidine and nifedipine were prepared by dissolving the compounds in DMSO. After baseline measurements, quinidine and nifedipine were added to each of the HD-MEA culture medium to reach final concentrations of 1 *μ*M and 100 nM, respectively. Postdose recordings were obtained 30 min after the drug administration to allow for equilibration of the compounds in the culture medium.^48^ The experiments were performed using the following electroporation parameters: rectangular pulses of 1 V peak-to-peak amplitude (Vpp), 200 *μ*s phase duration, 1 ms interpulse interval (IPI), 25,000 pulses simultaneously delivered through each of 200 electrodes of 4 *μ*m diameter. The used parameters are listed in Table 1, which also includes stimulation parameters used in prior studies.

### Data Analysis

Intracellular-like signal waveforms after electro-porations were analyzed after adjustment for the amplifier offsets. The waveforms were preprocessed with custom-made MATLAB-based scripts. Three consecutive waveforms, taken either 9 s after poration end or after channel stabilization, were averaged to obtain a single waveform per channel. We considered measured waveforms as intracellular-like signals if (i) the peak amplitude of the waveform was larger than 500 *μ*V, and (ii) the peak width was larger than 25 ms. The corresponding AP waveforms were normalized in amplitude since intracellular-like signals obtained by HD-MEAs were attenuated nonuniformly on each electrode depending on cell–electrode sealing resistances or membrane pore sizes.^14,29,32,42^ The AP parameters used for analysis in this study are displayed in Figure 3a. A two-sided Mann-Whitney *U* test was used to compare drug responses to baseline control experiments with a significance level of 0.05. Analyses then yielded alterations or signal changes as mean value ± standard deviation.

### Immunofluorescence Staining and Confocal Imaging

Cells on the HD-MEAs were fixated and prepared for immunostaining following a published protocol.^49^ After membrane permeabilization and blocking of nonspecific bindings, the samples were incubated with primary antibodies and, thereafter, with secondary antibodies. The used primary and secondary antibodies are listed in Table 2. To take images of immunostained cells on the HD-MEA chips, we used a Nikon NiE upright confocal microscope equipped with a Yokogawa W1 spinning-disk system.

### Scanning Electron Microscopy

Pt-black nanostructures on the HD-MEA electrodes were imaged using an FEI Helios NanoLab DualBeam 650 system after sputter-coating with 25 nm of gold. CMs on the HD-MEAs were fixated with IC Fixation Buffer at room temperature for 15 min and dehydrated by applying graded ethanol series. Subsequently, the sample was dried in a critical-point drier and sputter-coated with 5 nm of iridium. Focused-ion-beam-assisted sample cross section and images were acquired with a Xenon FIB-SEM TESCAN SOLARIS X system.

## Results And Discussion

### Transient Intracellular Access and Recovery of Cell Membrane after Electroporation

This study was aimed at achieving transient intracellular access on-demand to CMs so that intracellular-like signals could be obtained multiple times within a longer time span without damaging the CMs. Access was enabled by the electrodeposited dendritic Pt-black nanostructures on the electrodes of the HD-MEA (Figure 1a). The CM plasma membrane could be transiently opened through electroporation, intracellular-like signals were then obtained, and the cell membrane nanopores resealed afterward. Recordings from iPSC-derived CMs on HD-MEAs showed clear extracellular signals before electroporation. After applying the series of voltage pulses to the electrodes, the cellular signal waveforms transited from extracellular to intracellular-like shapes (Figure 1b) due to transient poration.^50^ Over time, the signal amplitudes of intracellular-like signals decreased, the signal waveforms showed a mix of intracellular-like and extracellular shapes, and the signal characteristics transited back to extracellular signals, as the membrane nanopores presumably resealed.

Porous and nanostructured Pt-black grown on electrodes entailed a low impedance and good electrical coupling between the electrodes and CM membranes. Presumably, Pt-black provided nanoscale protrusions, at which—upon applying poration protocols—high electrical field strengths locally occurred^51^ that then led to local membrane opening. Comparably long intracellular measurements were obtained using 5.5 × 9.3 *μ*m^2^ rectangular electrodes and 10,000 poration pulses featuring 2 Vpp amplitude, 1 ms phase duration, and 1 ms IPI (Figure S1a). The longest duration of intracellular-like recording—before a mix of intracellular and extracellular waveforms appeared—amounted to 75 min (Figure 1c). AP waveforms obtained every 10 min during intracellular recordings retained their shape after amplitude normalization (Figure S1b). With shorter pulses at a higher frequency (2 Vpp, 50 *μ*s phase duration, 6000 pulses), we could achieve an AP amplitude as high as 44.7 mV (Figure S1c). Upon re-applying the same voltage pulses to the same electrode after 2 h of recovery, a maximum AP amplitude of 41.9 mV was obtained. The third intracellular access after 2 h yielded intracellular-like signals of 28.6 mV amplitude. Such signal amplitudes are comparable to or larger than previously reported signal amplitudes obtained with dedicated 3D nanostructures, or by using optoporation methods on planar Pt-black-covered electrodes to record from iPSC-derived CMs.^26–28,33,52,53^

This finding suggests that the high porosity and roughness of Pt-black electrodes (Figure 1a), which can be achieved using a simple electroplating method,^46,54^ provides sufficiently low electrode impedance^55^ and good enough electrical coupling between cell membrane and electrode.^29^

### Simultaneous Electroporation and Whole-Cell Current Clamp Patch Measurements

We also performed simultaneous intracellular recordings on the HD-MEA with a patch electrode. While we conducted current clamp measurements with the patch electrode, we electroporated the same cell with a 4 *μ*m diameter electrode located underneath the cell using the following poration parameters: 5000 pulses of an amplitude of 1.6 Vpp, 200 *μ*s phase duration with 1 ms IPI. The electrode offset was adjusted after the stimulation in case that the HD-MEA amplifier was saturated (Figure 1d). We measured intracellular signals in two CMs (cell A, cell B), while both CMs continued beating during and after the electroporation (Figures 1d and S2a). After the electro-poration, the AP amplitude of the patch clamp measurement in the current clamp (IC) mode decreased significantly, from 96.9 to 62.2 mV in cell A (Figure 1d) and from 97.4 to 72.9 mV in cell B (Figure S2a). The reduction in AP amplitude of cell A (35.8%) was larger than that of cell B (25.2%), while HD-MEA recordings showed larger AP amplitudes in cell A after electroporation. One possible explanation is the size difference of nanopores that transiently opened at the cell–electrode interface. The larger the nanopores at the interface are, the more current leaks through the pores, which results in lower voltage signal amplitudes in the patch recordings upon membrane depolarization of the cells.^33^ This reasoning is further supported by the observation that the AP amplitude gradually increased in the patch recording while it decreased in the HD-MEA recording for both cells (Figures 1d and S2a) upon nanopore resealing with time.

### Optimization of Electroporation Parameters

**Figure 2:**
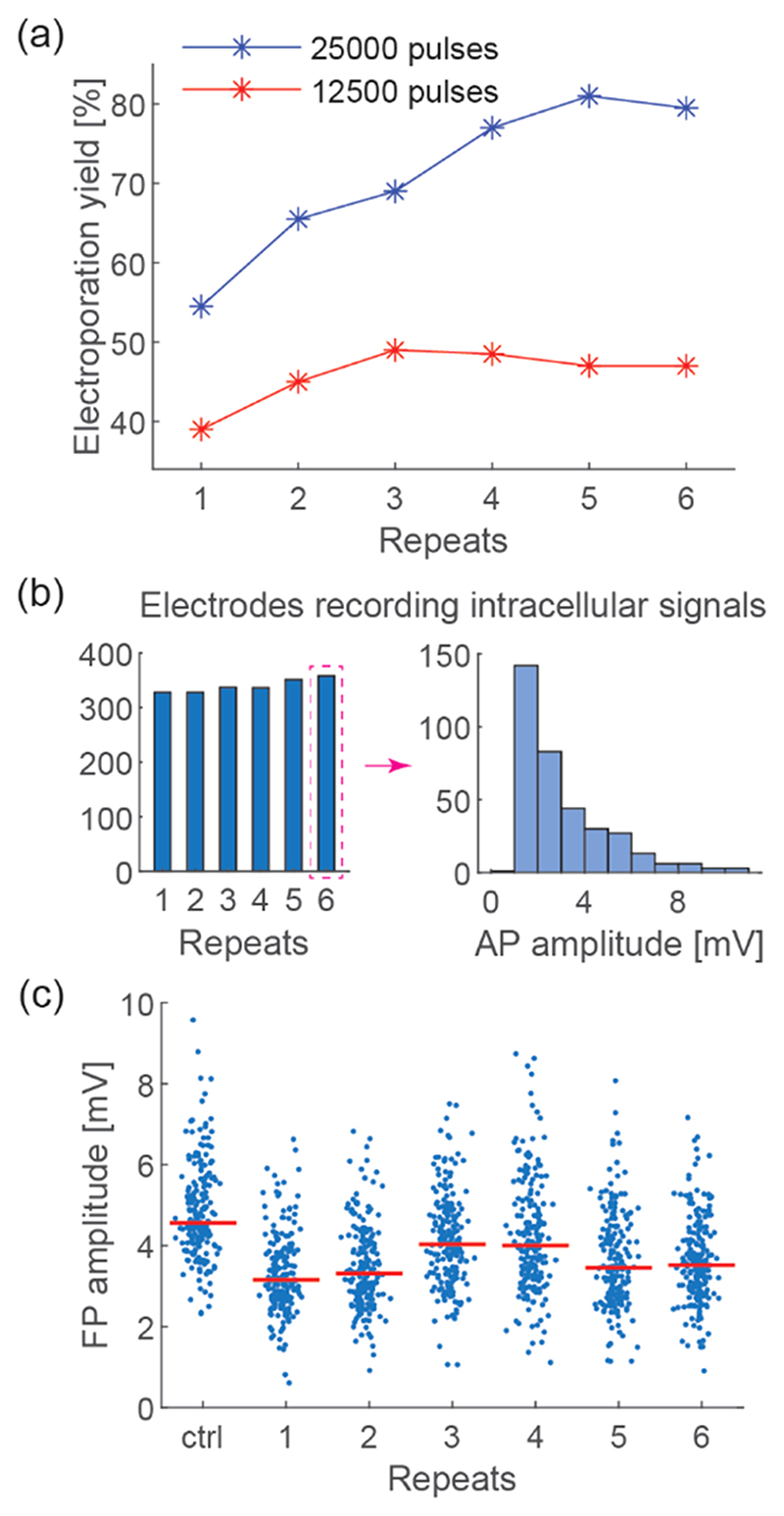
Optimization of electroporation parameters. (a) Electro-poration success rate at each repeat after 25,000 and 12,500 pulses had been delivered. Electroporation pulses were applied to 200 electrodes in parallel on two separate HD-MEA chips. (b) Number of electrodes that recorded intracellular signals after applying electro-poration pulses to 500 electrodes (left) and a histogram showing the distribution of AP amplitudes after the 6th electroporation (right). (c) Evolution of FP amplitudes before and after repeated electroporations using 200 standard 5.5 × 9.3 *μ*m^2^ rectangular electrodes. Each data point represents one electrode. Red bars indicate median values.

It is of critical importance to find electroporation parameters that yield only brief and transient access to intracellular-like signals, while the procedure remains as gentle on the cell as possible. Irreversible damage to cells has to be avoided so that the cells can recover before the next poration. The main focus of the optimization process was on attaining high electroporation yield with minimal impact on cellular behavior and signaling. First, the effects of voltage pulse amplitude, phase duration, and overall poration duration were investigated. We found that the electroporation yield increased with longer phase durations (Figure S3a) and higher poration voltages (Figure S3b). When the number of pulses was reduced by 50%, which decreased the total poration duration by 50%, the electroporation yield also significantly decreased throughout all repetitions (Figure 2a). All experimental conditions were tested in separate cultures to exclude potential effects of repeated electro-porations. Higher voltage, longer pulse phases, and longer overall poration duration (larger number of pulses and longer pulse phase durations) delivered higher charge density to the cell membrane so that larger or more nanopores may have been created in the membrane. Consequently, the poration yield was increased.

**Figure 3:**
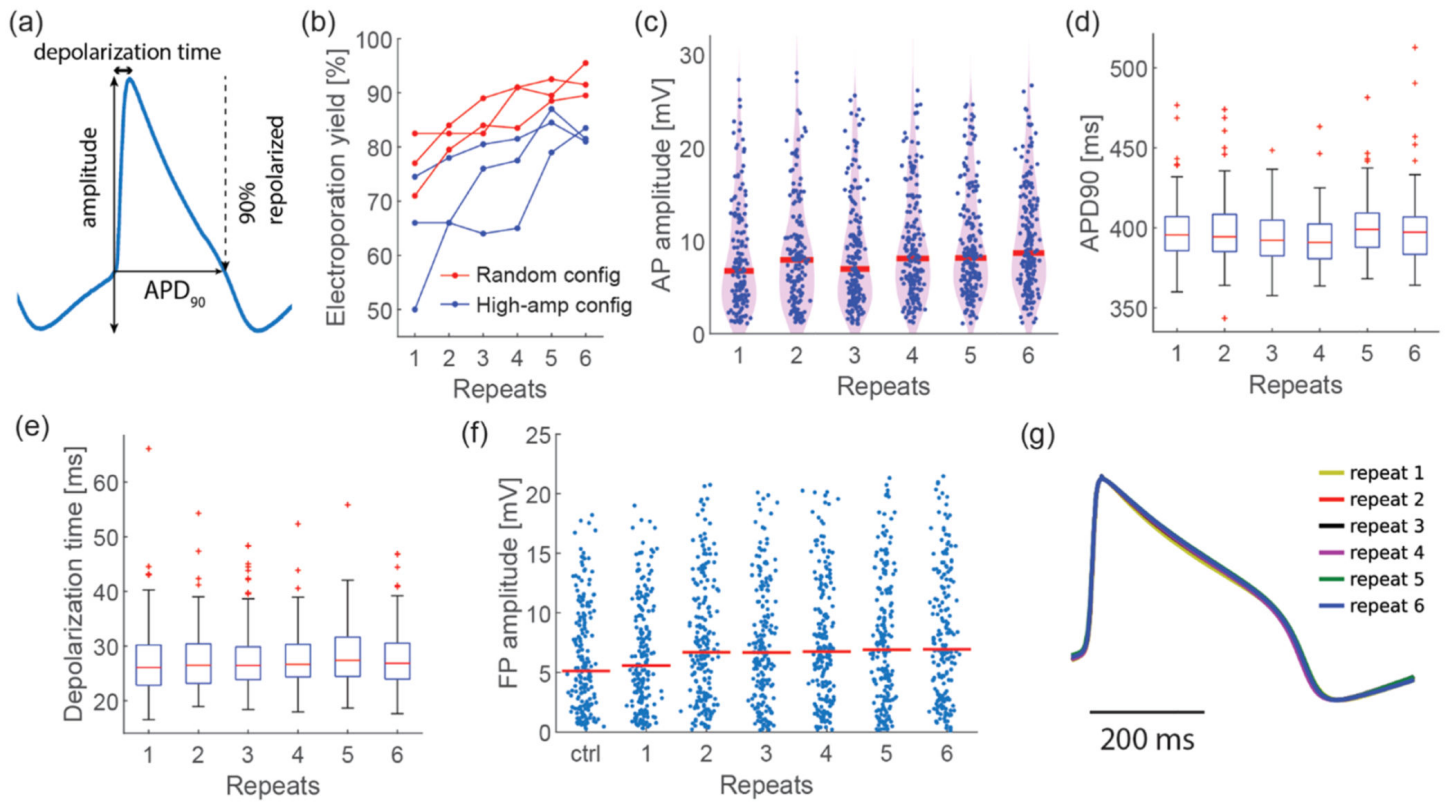
Electroporation with optimized parameters allows for repeated intracellular access to iPSC-derived CMs at high yield without damage. (a) Representative intracellular-like AP waveform and definition of characteristic AP parameters. (b) Electroporation yield at each repeat after sending voltage pulses to random configurations and high-amp configurations (*N* = 3 separate cultures per condition). (c–e) AP parameters extracted from intracellular-like recordings (*N* = 154, 168, 178, 182, 185, and 183 cells for repeats 1–6). Electroporation pulses were sent to 200 electrodes. In the box plots, red bars indicate median values, bottom and top boundaries of the boxes indicate the 25th and 75th percentiles, respectively; whiskers show the data range except for outliers, which are indicated by red crosses. Red bars in the violin plot represent median values. (f) Evolution of extracellular FP amplitudes before and after repeated electroporations using 200 circular 4 *μ*m diameter electrodes. Each data point represents one electrode. Red bars indicate median values. (g) Overlay of 6 AP waveforms after amplitude normalization, each of which is an average AP waveform of 154, 168, 178, 182, 185, and 183 cells for repeats 1–6; repetition interval was 1 h.

To enable large-scale drug screening assays, we recorded intracellular-like signals with hundreds of electrodes in parallel in a given CM culture. It is theoretically possible to send electroporation pulses and record signals from up to 990 electrodes in parallel with our HD-MEA system. However, connecting multiple electrodes to a stimulation unit decreases the slew rate of the poration voltage pulses due to increased capacitance, which may affect the electroporation yield. To elucidate this effect, we applied the same voltage pulses to 200 and 500 electrodes in separate CM cultures. The overall number of electrodes, which recorded intracellular-like signals (AP amplitude > 1 mV, Figure 3a) after electroporation, was higher when 500 electrodes were simultaneously used (Figure 2b), although the poration success rate was higher upon using 200 electrodes (Figure 3b). The measured AP amplitudes after poration were lower for using 500 electrodes in comparison to using 200 electrodes (Figures 2b and 3c), most likely due to increased capacitive loading at the stimulation units, which resulted from connecting more electrodes. The AP duration at 90% repolarization (APD90) and depolarization time remained consistent throughout 6 electroporations also for using 500 electrodes (Figure S3c,d). These results demonstrate that it is possible to repeatedly obtain simultaneous intracellular-like recordings at more than 300 locations without significant waveform changes (APD90 and depolarization time). This result is comparable to or better than that reported for using state-of-the-art techniques.^26,27,36,56^

Next, we investigated whether the spatial distribution or selection of the poration electrodes may affect the electro-poration success rate. We applied two electrode selection strategies. In the first approach, we used extracellular signal amplitudes as a criterion by assuming that a large extracellular signal amplitude could originate from a high sealing resistance between cell membrane and electrode. In the second approach, we applied random selection to have a more or less unbiased distribution of poration electrodes. After scanning the electrical activity across the HD-MEA, 200 electrodes were selected, either based on extracellular signal amplitude (“high-amp config”) or on random selection from regions where cellular activities were detected (“random config”, Figure S2b). Poration parameters included 1 Vpp amplitude, 200 *μ*s phase duration, 1 ms IPI, and 25,000 pulses. We found that electroporations performed with randomly selected electrodes (“random config” method) featured a higher yield compared to using the “high-amp config” method (Figure 3b). This finding is a consequence of the switch matrix scheme that routes electrodes to stimulation/poration units.^16^ A random selection of electrodes entails a more even distribution of poration electrodes over the 32 stimulation/poration units, while using the “high-amp config” may lead to uneven and skewed electrode distribution across the available stimulation units, which may result in heavy loading of a few stimulation units. Consequently, the results obtained using a “high-amp config” may greatly vary from preparation to preparation, while those achieved using a random selection are more stable and allow for better comparison.

Thereafter, we investigated the influence of the electrode shape and size. We assumed that small and round-shaped electrodes would deliver a more focused electrical poration pulse, which would enable targeted electroporation at a lower voltage. Previous studies had shown that Pt-black deposition significantly lowered the electrode impedance^54^ and decreased the electrode-size-dependent signal-attenuation to less than 2% for 11–86 *μ*m^2^ area electrodes.^57^ Therefore, we used HD-MEAs with circular electrodes of 4 *μ*m diameter (electrode area: 12.6 *μ*m^2^, Figures 1a and S2c) in our study and compared the results with those obtained with standard 5.5 × 9.3 *μ*m^2^ rectangular electrodes^16^ (electrode area: 50.7 *μ*m^2^). The use of 4 *μ*m diameter electrodes entailed significantly higher poration yield than that of standard electrodes for the same electro-poration pulses (1 Vpp, 200 *μ*s phase duration, 1 ms IPI, 25,000 pulses, Figures 3b and S3a). To reach a poration yield comparable to that of 4 *μ*m diameter electrodes, the stimulation voltage amplitude had to be increased to 1.4 Vpp for standard-size electrodes (Figure S3b). However, at this comparably high stimulation voltage, extracellular field potential (FP) amplitudes decreased by 30.3 ± 16.8% after the first electroporation and by 27.1 ± 19.6% after two electroporations even for phase durations as short as 50 *μ*s (Figure 2c). The experiments evidenced that 4 *μ*m diameter circular electrodes enabled us to perform more gentle electroporations at a higher success rate in comparison to standard-size electrodes.

Next, we studied the effects that repeated electroporations may have on the electrophysiology of iPSC-derived CMs. Electroporations were repeated on the same cells six times at 1 h intervals using the following stimulation parameters, selected according to the aforementioned optimization process: 1 Vpp, 200 *μ*s phase duration, 1 ms IPI, 25,000 pulses applied through 200 electrodes of 4 *μ*m diameter. Interestingly, the poration success rate increased across repetitions in contrast to a recent study showing a decrease over time.^27^ Presumably, repeated and gentle stimulations rendered the plasma membrane easier to open without killing the respective cells. The percentage of synchronously beating cells did not deteriorate during the six repeats (Figure S3e), suggesting that the electroporation did not negatively influence or suppress the electrical activity of CMs. Waveform characteristics, like APD90 and depolarization time, remained constant throughout repeated electroporations (Figure 3d, e). Moreover, extracellular signals measured before and after each electroporation showed that FP amplitudes did not decrease due to electroporations (Figure 3f). An overlay of averaged AP waveforms of six poration repeats (Figure 3g) demonstrates that there was no perceivable change in AP waveforms upon repeated electroporation. In addition, the AP amplitudes did not exhibit any sign of degradation throughout repeated electroporations (Figure 3c). All of these results indicate that the repeated electroporations were not hazardous to CMs and did not alter electrophysiological features, which is important to avoid misinterpretation of pharmacological responses during cardiotoxicity testing.

### Pharmacological Investigations

Following the optimized electroporation protocol, 200 electrodes were randomly selected in the active region of the HD-MEA to measure drug responses of CMs. One day before pharmacological experiments, iCell Maintenance Medium was replaced by serum-free CM culture medium. After three baseline measurements, compounds were added to the culture medium on the HD-MEAs.

**Figure 4:**
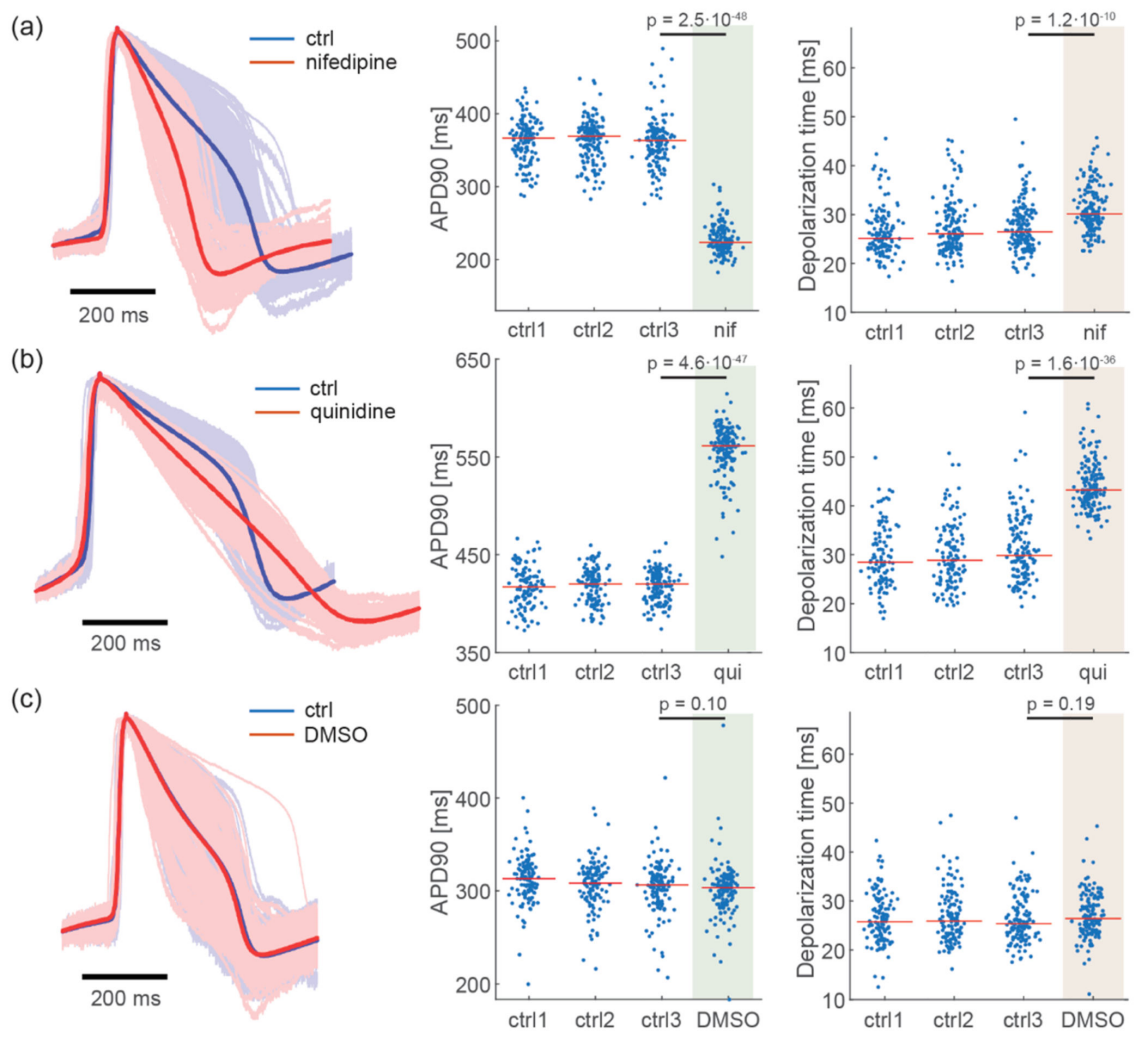
Pharmacological effects on intracellular-like AP waveforms. APD90 and depolarization time upon exposure of iPSC-derived CMs to (a) nifedipine, (b) quinidine, and (c) DMSO (vehicle control). AP waveforms of the 3rd baseline measurement (ctrl3) and upon drug exposure are overlaid in the left column. The thick-line waveforms in strong blue and red color have been obtained by averaging all individual blueish and reddish amplitude-normalized waveforms attained from array electrodes. APD90 shortening by nifedipine and prolongation by quinidine are shown in the center column. Drug effects on the depolarization time (almost unchanged for nifedipine and an increase for quinidine) are shown in the right column. DMSO, which was used as drug vehicle, did not affect the AP parameters at up to 0.1% concentration. Each data point represents the respective value obtained from one recording electrode. *p*-Values (two-sided Mann–Whitney *U* test) are displayed in each dot plot.

Nifedipine is an L-type Ca^2+^ channel blocker that is known to shorten APDs and cause triangularization of AP shapes.^13^ Upon administration, nifedipine induced a marked decrease of APD90s of iPSC-derived CMs (Figure 4a) as reported previously.^13,58^ Compared to the last baseline measurement, APD90 decreased by 36.9 ± 5.3% (*N* = 143, *p* < 0.05, two-sided Mann-Whitney *U* test). On the other hand, nifedipine increased the depolarization time only slightly (15.5 ± 18.9%, *N* = 143, *p* < 0.05), which also is in good agreement with previous studies.^13,58,59^

Quinidine is a Class 1a antiarrhythmic drug, which is known to block Na_v_1.5 and human ether-à-go-go-related gene (hERG) channels.^60–62^ Consistent with previous reports, quinidine induced significant prolongation of APD90s and an increase in the depolarization time (Figure 4b). After the quinidine administration, APD90s increased by 32.7 ± 4.9% (*N* = 139, *p* < 0.05) with respect to the last baseline measurement. Depolarization times increased by 46.4 ± 30.8% (*N* = 139, *p* < 0.05).

Since nifedipine and quinidine were dissolved in DMSO, we performed vehicle control experiments to test if 0.1% of DMSO has any effects on APD90 or depolarization time. We found that 0.1% of DMSO did not affect APD90s and depolarization times of iPSC-derived CMs (Figure 4c, *p* > 0.05), suggesting that the observed changes in APD90 and depolarization time were caused by the added drugs and not the vehicle DMSO.

The drug experiments with nifedipine and quinidine, whose effects on AP parameters are well characterized, demonstrate that our electroporation method with the HD-MEA reliably and reproducibly captures pharmacological effects on CM electrophysiology. Our results are in line with previous reports on the effects of nifedipine and quinidine.^13,33^ Nifedipine and quinidine caused modulations in APD90 and depolarization time, which reflected the blocking effects of Na^+^, Ca^2+^, and K^+^ channels.^13,33,34^ The stability of intracellular-like and extracellular electrophysiological parameters, measured throughout repeated electroporations without compound exposure (Figure 3), provides clear evidence that the observed waveform alterations were induced by the drugs and not by the electroporation itself. The possibility to perform multiple baseline measurements with preserved key AP and extracellular parameters (Figure 3) in between pharmacological experiments increases the reliability of the corresponding assay results.

## Conclusions

Cardiotoxicity is a serious off-target effect that must be investigated before a pharmaceutically active compound can be approved for patient use. The patch clamp technique has been considered the gold standard to analyze changes in electro-physiological signaling upon drug administration. However, the complexity of the classical patch clamp technique and its low-throughput limit applicability to cardiotoxicity testing in the pharmaceutical industry. The use of automated patch clamp is only applicable to cells in suspension.^2,40^ Previous studies have demonstrated that electroporation enables intracellular access to CMs.^27,28,43^ Obtaining reliable intracellular signals is critical for cardiotoxicity testing, as alterations in signal waveforms arising from the cell opening process itself could lead to a misinterpretation of drug effects.

Here, we reported on a reliable method—a combination of human iPSC technology and HD-MEAs—that could be used as a drug-testing platform to predict cardiotoxicity effects on human cells. The obtained data demonstrate that—by applying poration voltages that were somewhat lower than in previous studies^26,27^—intracellular-like recordings could be obtained repeatedly with minimal damage to the cells. Moreover, drug effects on Na^+^, Ca^2+^, and K^+^ channels could be investigated in intact cultures of interconnected CMs, which are physiologically more representative than individual cells in suspension. The possibility to measure intracellular-like signals from hundreds of CMs—in parallel and repeatedly—provides statistical power and information on the reproducibility and bandwidth of drug-induced effects under realistic tissue conditions, which is—in our view—a decisive asset in cardiotoxicity screening. Long-term stress effects on the cells that may be caused by repeated electroporations over several days still remain to be investigated. Future work may include developing 24-well or 96-well HD-MEA technologies to improve the assay throughput so that cardiotoxicity testing of multiple compounds can be performed in parallel. Moreover, the technique may be applicable to tissue slices and 3D organoids to obtain intracellular-like signals of cells at or near the surface. It can also potentially be used for personalized drug testing with patient-iPSC-derived CMs, which carry genetic variants associated with arrhythmogenic cardiomyopathies and channelopathies.

## Supplementary Material

Supplement The Supporting Information is available free of charge at https://pubs.acs.org/doi/10.1021/acssensors.2c01678. High-quality electrical coupling between Pt-black electrodes and CM membrane, simultaneous patch clamp and HD-MEA recordings from another cell (cell B), examples for electrode selection, confocal images of immunostained CMs, impact of stimulation voltage phase duration and amplitude on electroporation yield, and extracellular and intracellular signal features throughout repeated electroporations (PDF)

## Figures and Tables

**Table 1:** Comparison of Stimulation Parameters with Those of Prior Electroporation Studies on Planar MEAs

stimulation parameters	Hayes et al.^26^	Jans et al.^27^	Edwards et al.^28^	this study (after optimization)
amplitude	±1 V biphasic	±1.65 V biphasic	1 V^*a*^	±0.5 V biphasic
phase duration	60 *μ*s	10 ms	1 ms	200 *μ*s
poration duration	10 min	-^*b*^	30 s	35 s

a Pulse shape not specified.

b Not specified.

**Table 2:** Primary and Secondary Antibodies Used

antibody	target	dilution	supplier	Cat.
Hoechst	DNA	1:500	Thermo Fisher Scientific	H3570
anti-cardiac troponin T	cardiac troponin T	1:200	Abcam	AB45932
anti-sarcomeric alpha actinin	sarcomeric alpha actinin	1:200	Abcam	AB9465
anti-mouse, Alexa Fluor 647	mouse IgG	1:500	Thermo Fisher Scientific	A-31571
anti-rabbit, Alexa Fluor 488	rabbit IgG	1:500	Thermo Fisher Scientific	A-21206

## Abbreviations

HD-MEA: high-density microelectrode array
CHO: Chinese hamster ovary
HEK293: human embryonic kidney cell line
APC: automated patch clamp
CMOS: complementary metal-oxide semiconductor
iPSC: induced pluripotent stem cell
CM: cardiomyocyte
DMSO: dimethyl sulfoxide
SEM: scanning electron microscopy
APD: action potential duration
IPI: interpulse interval
FP: field potential
AP: action potential

## References

[1] Benjamin EJ, Virani SS, Callaway CW, Chamberlain AM, Chang AR, Cheng S, Chiuve SE, Cushman M, Delling FN, Deo R, De Ferranti SD (2018). Heart Disease and Stroke Statistics—2018 Update: A Report From the American Heart Association. Circulation.

[2] Bell DC, Fermini B (2021). Use of Automated Patch Clamp in Cardiac Safety Assessment: Past, Present and Future Perspectives. J Pharmacol Toxicol Methods.

[3] Sallam K, Li Y, Sager PT, Houser SR, Wu JC (2015). Finding the Rhythm of Sudden Cardiac Death. Circ Res.

[4] Singal PK, Iliskovic N (1998). Doxorubicin-Induced Cardiomyopathy. N Engl J Med.

[5] Avior Y, Sagi I, Benvenisty N (2016). Pluripotent Stem Cells in Disease Modelling and Drug Discovery. Nat Rev Mol Cell Biol.

[6] del Álamo JC, Lemons D, Serrano R, Savchenko A, Cerignoli F, Bodmer R, Mercola M (2016). High Throughput Physiological Screening of IPSC-Derived Cardiomyocytes for Drug Development. Biochim Biophys Acta, Mol Cell Res.

[7] May JE, Xu J, Morse HR, Avent ND, Donaldson C (2009). Toxicity Testing: The Search for an in Vitro Alternative to Animal Testing. Br J Biomed Sci.

[8] Sayed N, Liu C, Wu JC (2016). Translation of Human-Induced Pluripotent Stem Cells. J Am Coll Cardiol.

[9] Yamamoto W, Asakura K, Ando H, Taniguchi T, Ojima A, Uda T, Osada T, Hayashi S, Kasai C, Miyamoto N, Tashibu H (2016). Electrophysiological Characteristics of Human IPSC-Derived Cardiomyocytes for the Assessment of Drug-Induced Proarrhythmic Potential. PLoS One.

[10] Dunlop J, Bowlby M, Peri R, Vasilyev D, Arias R (2008). High-Throughput Electrophysiology: An Emerging Paradigm for Ion-Channel Screening and Physiology. Nat Rev Drug Discovery.

[11] Inayat S, Pinto LH, Troy JB (2013). Minimizing Cytosol Dilution in Whole-Cell Patch-Clamp Experiments. IEEE Trans Biomed Eng.

[12] Wei X, Zhuang L, Li H, He C, Wan H, Hu N, Wang P (2020). Advances in Multidimensional Cardiac Biosensing Technologies: From Electrophysiology to Mechanical Motion and Contractile Force. Small.

[13] Scheel O, Frech S, Amuzescu B, Eisfeld J, Lin KH, Knott T (2014). Action Potential Characterization of Human Induced Pluripotent Stem Cell-Derived Cardiomyocytes Using Automated Patch-Clamp Technology. Assay Drug Dev Technol.

[14] Xu D, Fang J, Zhang M, Wang H, Zhang T, Hang T, Xie X, Hu N (2021). Synchronized Intracellular and Extracellular Recording of Action Potentials by Three-Dimensional Nanoroded Electroporation. Biosens Bioelectron.

[15] Wäldchen S, Lehmann J, Klein T, Van De Linde S, Sauer M (2015). Light-Induced Cell Damage in Live-Cell Super-Resolution Microscopy. Sci Rep.

[16] Ballini M, Muller J, Livi P, Chen Y, Frey U, Stettler A, Shadmani A, Viswam V, Lloyd Jones I, Jackel D, Radivojevic M (2014). A 1024-Channel CMOS Microelectrode Array With 26,400 Electrodes for Recording and Stimulation of Electrogenic Cells In Vitro. IEEE J Solid-State Circuits.

[17] Yuan X, Schröter M, Obien MEJ, Fiscella M, Gong W, Kikuchi T, Odawara A, Noji S, Suzuki I, Takahashi J, Hierlemann A (2020). Versatile Live-Cell Activity Analysis Platform for Characterization of Neuronal Dynamics at Single-Cell and Network Level. Nat Commun.

[18] Dragas J, Viswam V, Shadmani A, Chen Y, Bounik R, Stettler A, Radivojevic M, Geissler S, Obien MEJ, Müller J, Hierlemann A (2017). In Vitro Multi-Functional Microelectrode Array Featuring 59 760 Electrodes, 2048 Electrophysiology Channels, Stimulation, Impedance Measurement, and Neurotransmitter Detection Channels. IEEE J Solid-State Circuits.

[19] Kato Y, Matoba Y, Honda K, Ogawa K, Shimizu K, Maehara M, Fujiwara A, Odawara A, Yamane CU, Kimizuka N, Ogi J (2020). High-Density and Large-Scale MEA System Featuring 236,880 Electrodes at 11.72*μ*m Pitch for Neuronal Network Analysis.

[20] Müller J, Ballini M, Livi P, Chen Y, Radivojevic M, Shadmani A, Viswam V, Jones IL, Fiscella M, Diggelmann R, Stettler A (2015). High-Resolution CMOS MEA Platform to Study Neurons at Subcellular, Cellular, and Network Levels. Lab Chip.

[21] Emmenegger V, Obien MEJ, Franke F, Hierlemann A (2019). Technologies to Study Action Potential Propagation with a Focus on HD-MEAs. Front Cell Neurosci.

[22] Blinova K, Dang Q, Millard D, Smith G, Pierson J, Guo L, Brock M, Lu HR, Kraushaar U, Zeng H, Shi H (2018). International Multisite Study of Human-Induced Pluripotent Stem Cell-Derived Cardiomyocytes for Drug Proarrhythmic Potential Assessment. Cell Rep.

[23] Ando H, Yoshinaga T, Yamamoto W, Asakura K, Uda T, Taniguchi T, Ojima A, Osada T, Hayashi S, Kasai C, Miyamoto N (2017). A New Paradigm for Drug-Induced Torsadogenic Risk Assessment Using Human IPS Cell-Derived Cardiomyocytes. J Pharmacol Toxicol Methods.

[24] Clements M (2016). Multielectrode Array (MEA) Assay for Profiling Electrophysiological Drug Effects in Human Stem Cell-Derived Cardiomyocytes. Curr Protoc Toxicol.

[25] Spira ME, Hai A (2013). Multi-Electrode Array Technologies for Neuroscience and Cardiology. Nat Nanotechnol.

[26] Hayes HB, Nicolini AM, Arrowood CA, Chvatal SA, Wolfson DW, Cho HC, Sullivan DD, Chal J, Fermini B, Clements M, Ross JD (2019). Novel Method for Action Potential Measurements from Intact Cardiac Monolayers with Multiwell Microelectrode Array Technology. Sci Rep.

[27] Jans D, Callewaert G, Krylychkina O, Hoffman L, Gullo F, Prodanov D, Braeken D (2017). Action Potential-Based MEA Platform for in Vitro Screening of Drug-Induced Cardiotoxicity Using Human IPSCs and Rat Neonatal Myocytes. J Pharmacol Toxicol Methods.

[28] Edwards SL, Zlochiver V, Conrad DB, Vaidyanathan R, Valiquette AM, Joshi-Mukherjee R (2018). A Multiwell Cardiac MGMEA Platform for Action Potential Recordings from Human IPSC-Derived Cardiomyocyte Constructs. Stem Cell Rep.

[29] Abbott J, Ye T, Krenek K, Gertner RS, Ban S, Kim Y, Qin L, Wu W, Park H, Ham D (2019). A Nanoelectrode Array for Obtaining Intracellular Recordings from Thousands of Connected Neurons. Nat Biomed Eng.

[30] Lin ZC, Xie C, Osakada Y, Cui Y, Cui B (2014). Iridium Oxide Nanotube Electrodes for Sensitive and Prolonged Intracellular Measurement of Action Potentials. Nat Commun.

[31] Dipalo M, Melle G, Lovato L, Jacassi A, Santoro F, Caprettini V, Schirato A, Alabastri A, Garoli D, Bruno G, Tantussi F (2018). Plasmonic Meta-Electrodes Allow Intracellular Recordings at Network Level on High-Density CMOS-Multi-Electrode Arrays. Nat Nanotechnol.

[32] Desbiolles BXE, de Coulon E, Bertsch A, Rohr S, Renaud P (2019). Intracellular Recording of Cardiomyocyte Action Potentials with Nanopatterned Volcano-Shaped Microelectrode Arrays. Nano Lett.

[33] Lin ZC, McGuire AF, Burridge PW, Matsa E, Lou HY, Wu JC, Cui B (2017). Accurate Nanoelectrode Recording of Human Pluripotent Stem Cell-Derived Cardiomyocytes for Assaying Drugs and Modeling Disease. Microsyst Nanoeng.

[34] Xie C, Lin Z, Hanson L, Cui Y, Cui B (2012). Intracellular Recording of Action Potentials by Nanopillar Electroporation. Nat Nanotechnol.

[35] Liu H, Bolonduro OA, Hu N, Ju J, Rao AA, Duffy BM, Huang Z, Black LD, Timko BP (2020). Heart-on-a-Chip Model with Integrated Extra- And Intracellular Bioelectronics for Monitoring Cardiac Electrophysiology under Acute Hypoxia. Nano Lett.

[36] Liu R, Lee J, Tchoe Y, Pre D, Bourhis AM, D’antonio-Chronowska A, Robin G, Lee SH, Ro YG, Vatsyayan R, Tonsfeldt KJ (2022). Ultra-Sharp Nanowire Arrays Natively Permeate, Record, and Stimulate Intracellular Activity in Neuronal and Cardiac Networks. Adv Funct Mater.

[37] Robinson JT, Jorgolli M, Shalek AK, Yoon MH, Gertner RS, Park H (2012). Vertical Nanowire Electrode Arrays as a Scalable Platform for Intracellular Interfacing to Neuronal Circuits. Nat Nanotechnol.

[38] Zhao Y, You SS, Zhang A, Lee JH, Huang J, Lieber CM (2019). Scalable Ultrasmall Three-Dimensional Nanowire Transistor Probes for Intracellular Recording. Nat Nanotechnol.

[39] Liu R, Chen R, Elthakeb AT, Lee SH, Hinckley S, Khraiche ML, Scott J, Pre D, Hwang Y, Tanaka A, Ro YG (2017). High Density Individually Addressable Nanowire Arrays Record Intracellular Activity from Primary Rodent and Human Stem Cell Derived Neurons. Nano Lett.

[40] Jahed Z, Yang Y, Tsai CT, Foster EP, McGuire AF, Yang H, Liu A, Forro C, Yan Z, Jiang X, Zhao MT (2022). Nanocrown Electrodes for Parallel and Robust Intracellular Recording of Cardiomyocytes. Nat Commun.

[41] Hu N, Xu D, Fang J, Li H, Mo J, Zhou M, Li B, Chen HJ, Zhang T, Feng J, Hang T (2020). Intracellular Recording of Cardiomyocyte Action Potentials by Nanobranched Microelectrode Array. Biosens Bioelectron.

[42] Hai A, Shappir J, Spira ME (2010). Long-Term, Multisite, Parallel, In-Cell Recording and Stimulation by an Array of Extracellular Microelectrodes. J Neurophysiol.

[43] Desbiolles BXE, de Coulon E, Maïno N, Bertsch A, Rohr S, Renaud P (2020). Nanovolcano Microelectrode Arrays: Toward Long-Term on-Demand Registration of Transmembrane Action Potentials by Controlled Electroporation. Microsyst Nanoeng.

[44] Schmid C, Wohnhaas CT, Hildebrandt T, Baum P, Rast G (2020). Characterization of ICell Cardiomyocytes Using Single-Cell RNA-Sequencing Methods. J Pharmacol Toxicol Methods.

[45] Zhao M, Tang Y, Zhou Y, Zhang J (2019). Deciphering Role of Wnt Signalling in Cardiac Mesoderm and Cardiomyocyte Differentiation from Human IPSCs: Four-Dimensional Control of Wnt Pathway for HiPSC-CMs Differentiation. Sci Rep.

[46] Ronchi S, Fiscella M, Marchetti C, Viswam V, Müller J, Frey U, Hierlemann A (2019). Single-Cell Electrical Stimulation Using CMOS-Based High-Density Microelectrode Arrays. Front Neurosci.

[47] Honda M, Kiyokawa J, Tabo M, Inoue T (2011). Electrophysiological Characterization of Cardiomyocytes Derived From Human Induced Pluripotent Stem Cells. J Pharmacol Sci.

[48] Millard D, Dang Q, Shi H, Zhang X, Strock C, Kraushaar U, Zeng H, Levesque P, Lu H-R, Guillon J-M, Wu JC (2018). Cross-Site Reliability of Human Induced Pluripotent Stem Cell-Derived Cardiomyocyte Based Safety Assays Using Microelectrode Arrays: Results from a Blinded CiPA Pilot Study. Toxicol Sci.

[49] Ronchi S, Buccino AP, Prack G, Kumar SS, Schröter M, Fiscella M, Hierlemann A (2021). Electrophysiological Phenotype Characterization of Human IPSC-Derived Neuronal Cell Lines by Means of High-Density Microelectrode Arrays. Adv Biol.

[50] Xu D, Mo J, Xie X, Hu N (2021). In-Cell Nanoelectronics: Opening the Door to Intracellular Electrophysiology. Nano-Micro Lett.

[51] Dipalo M, Caprettini V, Bruno G, Caliendo F, Garma LD, Melle G, Dukhinova M, Siciliano V, Santoro F, De Angelis F (2019). Membrane Poration Mechanisms at the Cell-Nanostructure Interface. Adv Biosyst.

[52] Cerea A, Caprettini V, Bruno G, Lovato L, Melle G, Tantussi F, Capozza R, Moia F, Dipalo M, De Angelis F (2018). Selective Intracellular Delivery and Intracellular Recordings Combined in MEA Biosensors. Lab Chip.

[53] Iachetta G, Colistra N, Melle G, Deleye L, Tantussi F, De Angelis F, Dipalo M (2021). Improving Reliability and Reducing Costs of Cardiotoxicity Assessments Using Laser-Induced Cell Poration on Microelectrode Arrays. Toxicol Appl Pharmacol.

[54] Boehler C, Stieglitz T, Asplund M (2015). Nanostructured Platinum Grass Enables Superior Impedance Reduction for Neural Microelectrodes. Biomaterials.

[55] Spira ME, Huang SH, Shmoel N, Erez H (2019). Advances in Neurobiology.

[56] Abbott J, Ye T, Qin L, Jorgolli M, Gertner RS, Ham D, Park H (2017). CMOS Nanoelectrode Array for All-Electrical Intracellular Electrophysiological Imaging. Nat Nanotechnol.

[57] Viswam V, Obien MEJ, Franke F, Frey U, Hierlemann A (2019). Optimal Electrode Size for Multi-Scale Extracellular-Potential Recording from Neuronal Assemblies. Front Neurosci.

[58] Ma J, Guo L, Fiene SJ, Anson BD, Thomson JA, Kamp TJ, Kolaja KL, Swanson BJ, January CT (2011). High Purity Human-Induced Pluripotent Stem Cell-Derived Cardiomyocytes: Electrophysiological Properties of Action Potentials and Ionic Currents. Am J Physiol: Heart Circ Physiol.

[59] Guo L, Qian J-Y, Abrams R, Tang H-M, Weiser T, Sanders MJ, Kolaja KL (2011). The Electrophysiological Effects of Cardiac Glycosides in Human IPSC-Derived Cardiomyocytes and in Guinea Pig Isolated Hearts. Cell Physiol Biochem.

[60] Blinova K, Stohlman J, Vicente J, Chan D, Johannesen L, Hortigon-Vinagre MP, Zamora V, Smith G, Crumb WJ, Pang L, Lyn-Cook B (2017). Comprehensive Translational Assessment of Human-Induced Pluripotent Stem Cell Derived Cardiomyocytes for Evaluating Drug-Induced Arrhythmias. Toxicol Sci.

[61] Navarrete EG, Liang P, Lan F, Sanchez-Freire V, Simmons C, Gong T, Sharma A, Burridge PW, Patlolla B, Lee AS, Wu H (2013). Screening Drug-Induced Arrhythmia Using Human Induced Pluripotent Stem Cell-Derived Cardiomyocytes and Low-Impedance Microelectrode Arrays. Circulation.

[62] Crumb WJ, Vicente J, Johannesen L, Strauss DG (2016). An Evaluation of 30 Clinical Drugs against the Comprehensive in Vitro Proarrhythmia Assay (CiPA) Proposed Ion Channel Panel. J Pharmacol Toxicol Methods.

