# Supplementary material for: Repeated and On-Demand Intracellular Recordings of Cardiomyocytes Derived from Human-Induced Pluripotent Stem Cells
Source: ACS Sens. Author manuscript; Available in PMC 2022 Oct 28. (PMC7613763; doi:10.1021/acssensors.2c01678)
Supplement: Supplement — The Supporting Information is available free of charge at https://pubs.acs.org/doi/10.1021/acssensors.2c01678. High-quality electrical coupling between Pt-black electrodes and CM membrane, simultaneous patch clamp and HD-MEA recordings from another cell (cell B), examples for electrode selection, confocal images of immunostained CMs, impact of stimulation voltage phase duration and amplitude on electroporation yield, and extracellular and intracellular signal features throughout repeated electroporations (PDF) [file EMS155059-supplement-Supplement.docx]

Supporting Information

**Repeated and On-Demand Intracellular Recordings of Cardiomyocytes Derived from Human Induced Pluripotent Stem Cells**

Jihyun Lee^1,*^, Tobias Gänswein^1^, Hasan Ulusan^1^, Vishalini Emmenegger^1^, Ardan M. Saguner^2^, Firat Duru^2,3^, Andreas Hierlemann^1,*^

1. Bio Engineering Laboratory, ETH Zurich, 4058 Basel, Switzerland

2. Cardiac Electrophysiology Division, University Heart Center Zurich, University Hospital Zurich, 8091 Zurich, Switzerland

3. Center for Integrative Human Physiology, University of Zurich, 8057 Zurich, Switzerland

*Corresponding authors: Jihyun Lee; Bio Engineering Laboratory, ETH Zurich, 4058 Basel, Switzerland; Phone: +41 (0)61 387 31 28; Email: jihyun.lee@bsse.ethz.ch, Andreas Hierlemann; Bio Engineering Laboratory, ETH Zurich, 4058 Basel, Switzerland; Phone: +41 (0)61 387 31 50; Email: andreas.hierlemann@bsse.ethz.ch


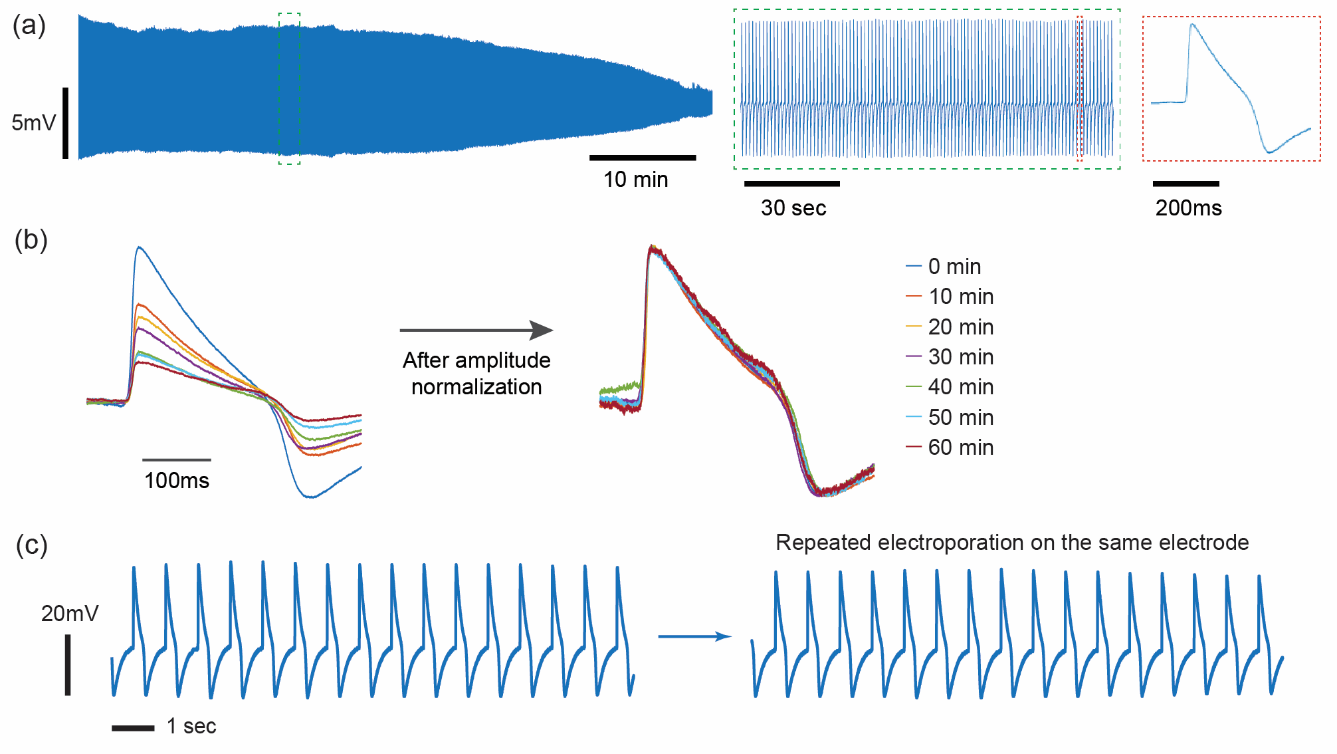


Figure S 1. High-quality electrical coupling between Pt-black electrodes and CM membranes. (a) Long intracellular-like recording and close-up images of AP waveforms. (b) Overlay of AP waveforms obtained between 0 to 60 min after the poration. (c) Intracellular-like recordings that exhibited large AP signal amplitudes were obtained repeatedly from the same cell.


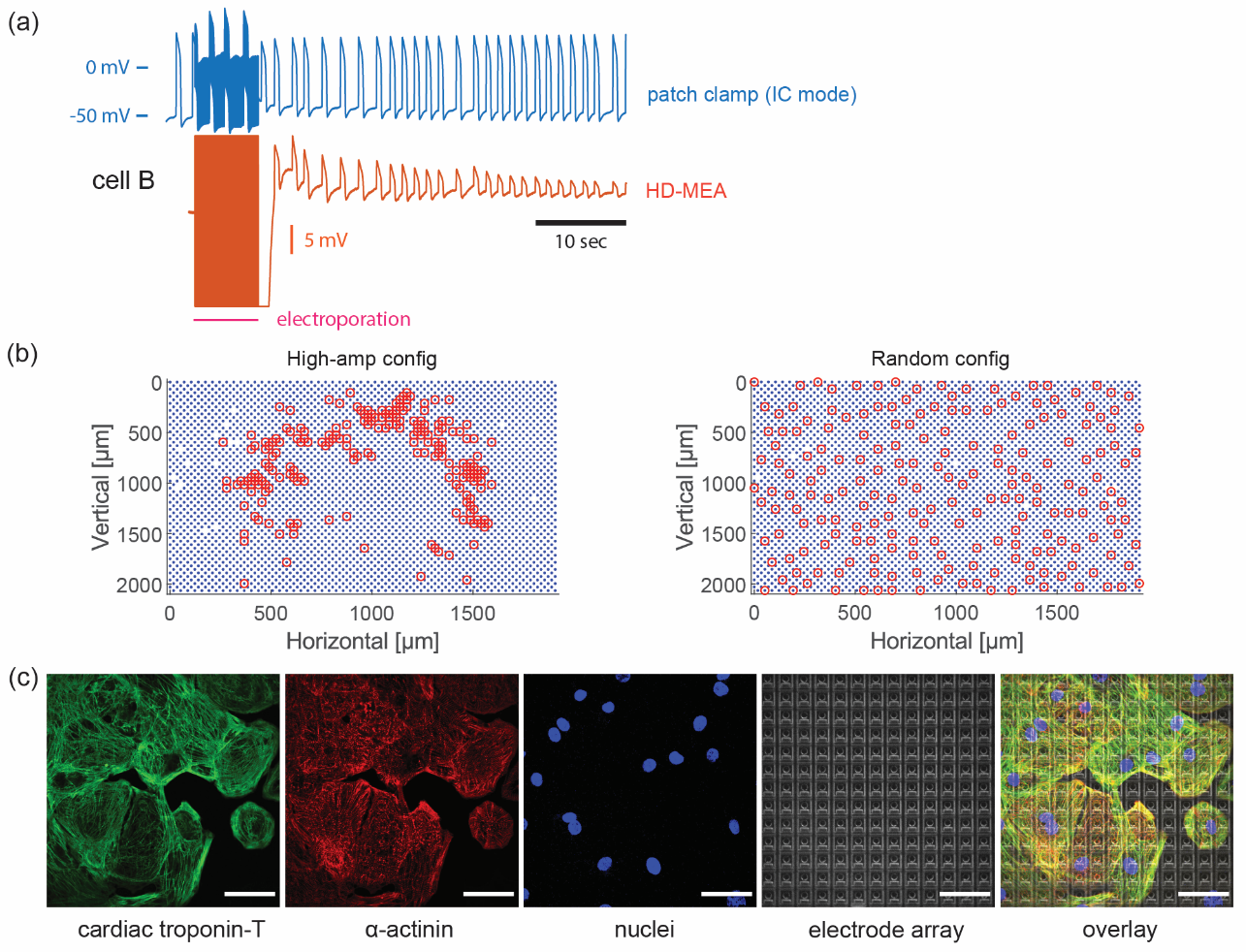


Figure S 2. (a) Simultaneous intracellular recordings by whole-cell patch clamp in the current-clamp (IC) mode and intracellular-like measurements by HD-MEAs after electroporation of the same cell (cell B). (b) Examples of two methods for electrode selection. Blue dots represent electrodes on which cellular electrical activities were detected. Red circles indicate the selected electrodes. (c) Confocal images of iPSC-derived CMs that have been immunostained on a HD-MEA with circular 4-µm-diameter electrodes. Images were taken using excitation wavelengths of 488 nm, 647 nm, 405 nm, and the brightfield mode (from left to right). Scale bar is 50 µm.


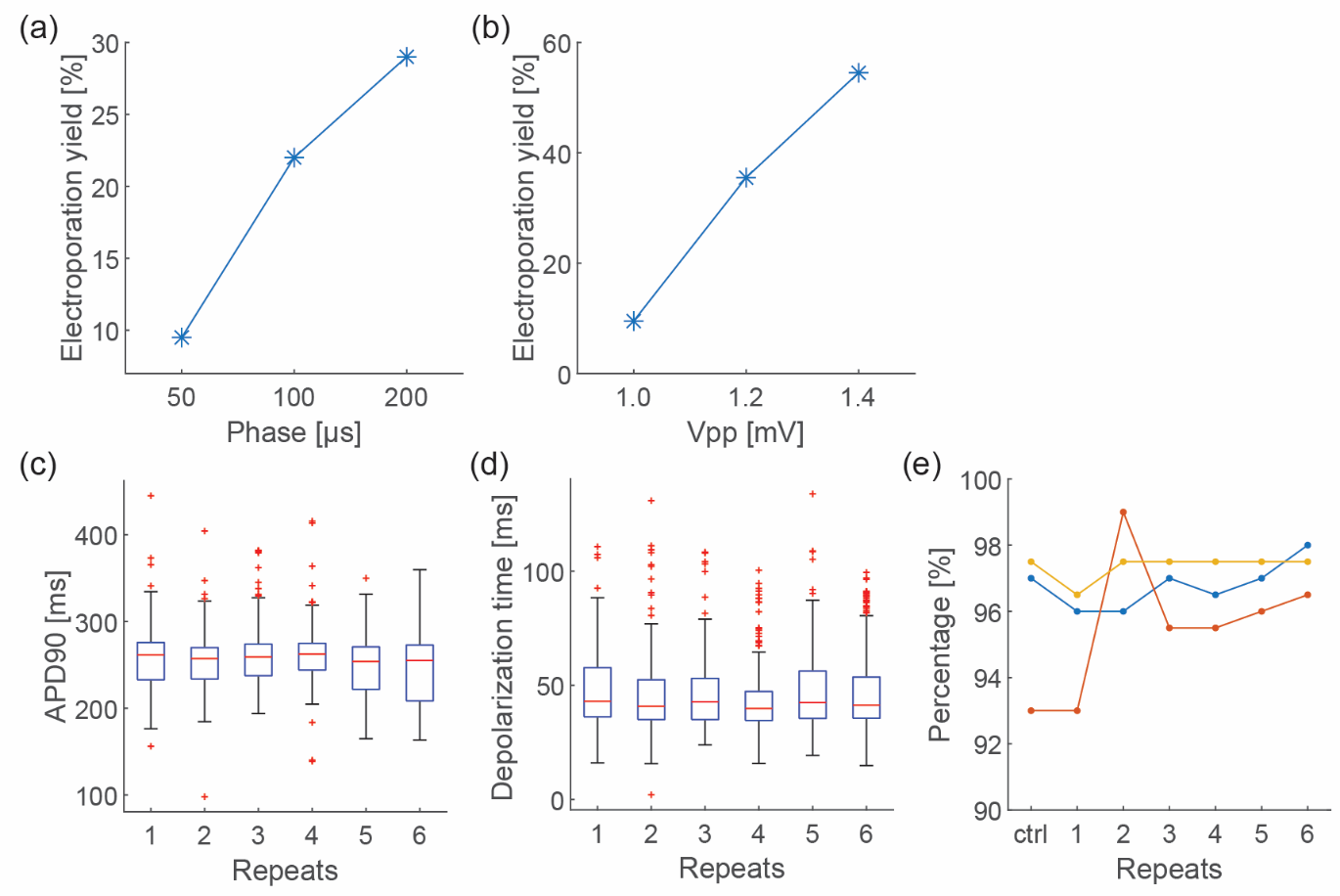


Figure S 3. (a) Electroporation yield for pulse phase durations of 50, 100, and 200 µs. Other parameters included 1 Vpp, 25000 pulses, 1ms IPI. (b) Electroporation yield at peak-to-peak voltage amplitudes of 1, 1.2, 1.4 Vpp. Other parameters included 50 µs phase duration, 25000 pulses, 1ms IPI. (c,d) APD90 and depolarization time calculated from intracellular-like recordings. Electroporation pulses were sent to 500 electrodes. The sample number N for each repetition is shown in Figure 2b. In the box plots, red bars indicate the median value, while the bottom and top boundaries of the boxes indicate the 25th and 75th percentile; the whiskers display the data range except for outliers that are indicated by red crosses. (e) Percentage of electrodes that detected synchronous beatings in extracellular measurements.
